# Analysis of a brief biodata scale as a predictor of job performance and its incremental validity over the Big Five and Dark Tetrad personality traits

**DOI:** 10.1371/journal.pone.0274878

**Published:** 2022-09-30

**Authors:** Pedro J. Ramos-Villagrasa, Elena Fernández-del-Río, Ángel Castro

**Affiliations:** 1 Department of Psychology and Sociology, Faculty of Labour and Social Sciences, University of Zaragoza, Zaragoza, Spain; 2 Department of Psychology and Sociology, Faculty of Social and Human Sciences, University of Zaragoza, Teruel, Spain; St John’s University, UNITED STATES

## Abstract

The collection of biographical information (biodata) through CVs and application forms has many advantages, namely easiness of collection, acceptable validity, less prone to faking, and the fulfilment of legal requirements. However, its systematic use among practitioners is scarce. Two of the mains reasons is the overlap with other constructs like personality and the lack of validated biodata scales in articles and public repositories. Aimed to fill this gap, García-Izquierdo and colleagues developed an 8-item scale able to generate positive applicant reactions, but they did not provide empirical evidence that their scale is able to predict job performance. The present paper was developed for this purpose, investigating the scale’s relationship with four different dimensions of job performance (i.e., task performance, contextual performance, counterproductive behaviors, and adaptive performance) and its incremental validity with respect to Big Five and Dark Tetrad personality traits. The study comprises 528 employees from different organizations (*M*_*age*_ = 39.51, *SD* = 14.25; 52.8% women, *M*_*experience*_ = 17.06, *SD* = 13.27) which voluntarily agreed to participate filling a questionnaire with the variables of interest. Results provide evidence of the predictive validity of the biodata scale in a multi-occupational sample; identify that these biodata contribute to predicting two specific types of job performance: contextual performance and adaptive performance; shows that a brief job-related biodata scale achieves results comparable to those of most personality traits in predictive models of job performance dimensions; and provide evidence of the incremental predictive validity of biodata over the Big Five and the Dark Tetrad. As a whole, these results provide support for the use of the scale in researcher and applied settings, and contributes to the advance the knowledge of biodata for personnel selection.

## Introduction

Recruitment, which can be defined as the process of attracting competent people to apply to a job offer [1], implies the collection and analysis of biographical information (biodata) of applicants [2, 3]. By analyzing biodata, practitioners can rule out applicants with inadequate job-fit, and gather information to use in combination with other evaluations that will be carried out later (interviews, tests, etc.) with the remaining candidates. With the digitalization of recruitment (e-recruitment), applicant screening using biodata is easier than ever, increasing the interest of researchers and practitioners about it [4].

Previous research highlighted that biodata has acceptable validity [3], are easy to collect from CV and application forms [5], are less prone to faking than other methods like personality inventories because may be based on verifiable information [6], and can ensure complying with legal requirements if its items demonstrate to be job-related [7]. However, the use of biodata among practitioners is considerably scarce [8]. One of the reasons is the lack of access to specific scales like research papers, repositories, etc. [9] Another issue is that previous research has found a considerable overlap with other constructs like cognitive ability and personality [3, 10]. This is due to broader considerations of biodata, including items related to opinions, attitudes, or general situations that become very close to personality scale items [11], losing the biographical character of biodata that differentiates it from other constructs. A solution to this problem is to focus only on past experiences, behaviors, and feelings in specific situations [12, 13], which is also related to recent calls to gather fair, legal, and bias-free information in personnel selection [3].

Even if these issues are considered in the development of biodata, the question of access to suitable biodata items remains open. Aimed at contributing in that sense, García-Izquierdo and colleagues [14] developed a set of biodata and they assess each item in terms of perception of predictive validity, fairness, and privacy. As a result, they proposed an 8-item scale able to generate positive applicant reactions. However, an important issue is pending: to demonstrate that their scale can predict job performance. The present paper was developed for this purpose, investigating the scale’s relationship with four different dimensions of job performance (i.e., task performance, contextual performance, counterproductive behaviors, and adaptive performance) in a multiocupational sample. Furthermore, the present research also investigates the degree to which biodata increases predictive validity over personality traits, including the Big Five and Dark Tetrad traits.

### Biodata and job performance

Biodata is based on behavioral consistency [14], referring to previous life experiences that seem to be related to present behavior [7, 15]. In that sense, several studies have found that biodata is associated with job performance [e.g., 16]. A recent meta-analysis reports correlations with overall performance ratings between .24 and .44 [13]. This data has been reviewed, suggesting an operational validity value of .35, which places biodata among the most recommendable personnel selection procedures [17]. But not all biodata should be considered equal: its validity depends on what we consider to be biodata and how the scale scoring is performing. Both issues will be discussed below.

Concerning what is biodata, previous research has found that biodata tends to overlap with other constructs like personality and cognitive ability [18], but even in this circumstance, biodata provides incremental validity [19]. Despite these positive results, any personal characteristic measured in personnel selection should demonstrate construct and content validity [20]. Recent research has suggested that biodata should be related to any of the following domains [13]: (a) mental capacity; (b) knowledge and procedural skills; (c) social skills; (d) agreeableness; (e) conscientiousness; (f) emotional stability and self-confidence; (g) openness to experience; (h) leadership; (i) academic achievement; (j) interests/preferences; (k) physical fitness; and (l) other specific experiences. Another issue related to validity is the content of the biodata items. It is recommended that it should be related to specific situations and contexts [9, 21] and based only on verifiable information [7].

However, what is missing in prior research [e.g., 13, 21–23] is to explore the relationship with performance dimensions. Previous authors have mainly focused on overall performance ratings (i.e., a single item measured workers’ behavior), construct-aligned performance ratings (i.e., related to a specific domain interest, like leadership), objective performance (i.e., effectiveness), training performance, and advancement potential. In other words, all criteria are related to task performance, while ignoring other relevant domains like contextual performance, counterproductive work behaviors, and adaptive performance [24, 25]. These dimensions have in common that they are behaviors displayed by the worker that contribute to organizational goals [26] but in different ways. Following Rotundo and Sackett [27], task performance is related to the production of goods and services, mainly as mentioned in job description; contextual performance comprises behaviors that contribute to the social and psychological environment; counterproductive behaviors are deviant behaviors with negative consequences at the individual or the organizational level; finally, adaptive performance has been recently proposed, and is described as behaviors displayed by workers to change according to job demands [28]. Identifying to what extent biodata functioning varies according to each performance dimension would help to optimize selection processes, making it easier to choose predictors as a function of the criterion of interest.

The other issue related to biodata predictive validity is biodata scoring. There are three different ways to estimate scores of biographical data [22]: rational, empirical, and quasi-rational. Rational scoring, which is based on literature review and experts’ criteria, reports a relationship with performance of .24 [13] but is easier to defend in case of litigation because the items are based on citable sources that can justify why to choose this biodata [7, 29]. Empirical scoring increases the relationship between biodata and the criteria to .48 [13] but is criticized for its “black box empiricism” [30], that is, the relevance of each biodata item is based on mathematical criteria only. The quasi-rational approach seems more suitable because it combines a rational development of biodata with an empirical one to weigh the items [30, 31]. Its functioning is not as good as the empirical one but is better than the rational one [32] and easier to explain [31]. Differences in scoring bring us back to the aforementioned issue concerning biodata availability. To increase the use of biodata among applicants, even among researchers, we need studies that share the results of biodata scales. The publication of biodata scales, especially at the item level [9] makes it easier to test the effect of different types of scoring, investigate their differential use in other types of samples, make adaptations to other countries, and more. García-Izquierdo and colleagues [14] contribute in that sense, but their study has some limitations that we need to overcome.

### The present study

The study developed by García-Izquierdo et al. [14] aimed to develop personnel selection biodata for managerial positions in the Spanish public administration, ensuring efficacy, transparency, and legality. They gathered data from two target groups: senior managers and applicants. The managers contributed to developing up to 26 biodata items, of which 15 were related to the quality of experience, 4 to managerial competencies, 3 to innovation and proactivity, 2 to social responsibility, and 2 to transparency. After that, managers and applicants assessed each item in three dimensions: perceived performance, fairness, and privacy. The result was 8 job-related items that achieved high scores, although none simultaneously in all three categories. Among its limitations, the authors recognized that the final biodata scale was only a proposal, and that confirmation is needed of its relationship with actual performance. The present study aims to fill this gap. We also want to test whether the scale can be applied to settings different than public management.

This study also aims to make further contributions to biodata research. Regarding its relationship with performance, we want to extend the research investigating whether biodata can predict not overall performance but its different dimensions (i.e., task performance, contextual performance, counterproductive behaviors, and adaptive performance) considering its multidimensional nature [24, 25].

In addition, we want to investigate the incremental validity of biodata over personality traits (Big Five and Dark Tetrad at the same time). To our knowledge, this is the first study to do so. Personality, conceptualized in the form of the Big Five traits (i.e., neuroticism, extraversion, openness, agreeableness, and conscientiousness) has an undeniable relevance in the work setting [33]. Its relationship with job performance has been demonstrated in several meta-analyses [17], pointing out a positive association with all traits except neuroticism. Regarding biodata, the meta-analysis by Speer and colleagues [13] shows a moderate-to-high overlap with Big Five traits but seems to be related to the convergence among domains. For example, biodata related to the social skills domain tends to be associated with extraversion.

Continuing with personality, in the last few years, interest in certain undesirable personality traits, namely the Dark Tetrad, has been growing in organizational contexts [34–37]. The Dark Tetrad comprises four traits [38]: narcissism, characterized by lack of empathy, attention-seeking, and sense of entitlement; Machiavellianism, based on manipulation and self-interest; subclinical psychopathy, related to the absence of remorse, antisociality, and lack of guilt; and sadism, which is related to the enjoyment of cruelty. Previous research has found that these traits have a different relationship with the job performance dimensions [34, 39]: narcissism is positively related to task, contextual and adaptive performance, Machiavellianism is positively associated with task performance, psychopathy is negatively related to task and adaptive performance, and sadism is negatively linked with task performance and positively related with counterproductive behaviors at work. Regarding the relationship with biodata, to the best of our knowledge, this is the first time that is considered together.

Last but not least, our study is also aimed to contribute to biodata scoring. The debate regarding biodata scoring is still ongoing. Although the evidence suggest that empirical biodata has better results in terms of predictive validity [17], there are concerns about its use in actual selection processes because some biographical data or the weight assigned by the mathematical model may be questionable from the point of view of inclusive personnel selection [20]. With the present research, we analyse the differential functioning of the same biodata scale depending whether the items are treated as rational or quasi-rational. In this line, Answering the call by Breaugh [9], we are going to provide full information about the biodata scale, including item-level information, weights for estimating the scale score, and full access to our data through an open access repository (see below).

## Method

### Participants and procedure

The study included 528 employees (*M*_*ag*e_ = 39.51, *SD* = 14.25; 52.8% women) from different organizations. Most of them were white-collar workers (68.56%), followed by blue-collar (28.03%) and military (1.14%). Twelve participants (2.27%) did not report their job. Average job experience was 17.06 years (*SD* = 13.27) and 25.8% of them held a position of responsibility among other workers.

Data were collected with non-probability sampling with the collaboration of trained university students, who distributed the questionnaire to the workers they knew. To perform our study, we follow the guidelines of the American Psychological Association (APA) Ethics Code (https://www.apa.org/ethics/code, sections 8 and 9). Specifically, participants were informed orally and in writing about: (1) study purposes; (2) the type of information to be collected from them; (3) how data would be treated; (4) that no personal information that allows identification was collected (e.g., names, address, emails); (5) their right to decline to participle or withdraw; (6) whom to contact for questions about the research or its results. The consent of participants was obtained orally. Workers voluntarily agreed to participate and were informed about the research objectives of this study and the confidentiality and anonymity of their responses. The open database are available at https://doi.org/10.17605/OSF.IO/WC5JU.

### Measures

#### Sociodemographic and work characteristics

We asked participants about their sex, age, job experience, and whether they held a position with responsibility among other workers.

#### Biodata

We used the 8 items developed by García-Izquierdo et al. [14]. Items 1 (“Have you held more than one position in public management?”) and 8 (“Do you think technology is a real help in public management?”) should be adapted to be used in any kind of company (i.e., Item 1: “Have you held more than one position in your organization?” and Item 8: “Do you think technology is a real help in organizational success?”). Scale items have a yes/no response format and are displayed in Table 1. According to Speer et al.’s [13] classification, this scale is multidimensional, with two items belonging to the openness to experience domain, two to the Leadership domain, and the remaining to conscientiousness, knowledge and procedural skills, Interest/preferences, and the Overall composite scale dimensions. It is also noteworthy that all items are job-related, and most of them (except for Item 8) are verifiable during the personnel selection process. Item 6, although it refers to leadership competences, includes competences that are related to performance in most jobs.

**Table 1:** Biodata items and domains to which they belong.

Biodata items	Domain according to Speer et al. (2021)
1. Have you occupied more than one post in your organization?	Overall composite scale
2. Have you made innovative improvements in your job?	Openness to experience
3. Have you conducted different projects?	Conscientiousness
4. Have you supervised teams consisting of five or more members?	Leadership
5. Have you successfully supervised teams?	Leadership
6. Do you believe you are sufficiently skilled in leadership competences (planning, negotiation, decision-making, communication, etc.)?	Knowledge and procedural skills
7. Do you consider yourself creative in problem-solving?	Openness to experience
8. Do you think technology is a real help in organizational success?	Interest/preferences

In our study, we used two different ways of scoring, rational and quasi-rational. Rational scoring was computed with the number of items scored as *yes*. Quasi-rational was estimated granting a different weight to each item according to its mean association with job performance. To develop the weights for quasi-rational scoring, we randomly split the sample into two subsamples of the same size (*N* = 264), one for calibration (Subsample 1) and the other for the study (Subsample 2). Table 1 shows the point-biserial correlations between items and the four dimensions of the criteria analyzed in the study. Weight was the mean value of the association of biodata items with job performance.

Both methods of scoring had lower internal consistency than was expected (α = .49 and α = .59, respectively). An item-level analysis (Table 2) led us to remove Item 1, which seems reasonable because it is the only biodata that does not have a significant association with any criteria. Thus, the 7-item version was used in this study (α = .63 for both scoring methods).

**Table 2:** Point-biserial associations between biodata items and performance.

Biodata item nº	Task performance	Contextual performance	Counterproductive behaviors	Adaptive performance	Weight in biodata scale
1	.073	.087	.019	-.056	0.007
2	.022	.283**	-.015	.162**	0.113
3	.096	.387**	-.082	.277**	0.170
4	.075	.306**	-.032	.166**	0.129
5	.087	.322**	.025	.186**	0.155
6	.170**	.350**	.045	.118	0.177
7	.080	.163**	.040	.116	0.280
8	.088	.042	-.017	.168**	0.268

*Note*.

*n* = 264 (Subsample 1).

** = *p* < .01.

#### Big Five personality traits

The Big Five was measured with the Spanish version of the NEO-FFI [40]. Each dimension is measured with 12 items which are rated on a 5-point Likert scale ranging from 1 (*strongly disagree*) to 5 (*strongly agree*). It measures neuroticism (α = .81), extraversion (α = .76), openness (α = .79), agreeableness (α = .70), and conscientiousness (α = .77). Total scores were computed as the sum of the scores of each dimension.

#### Dark Tetrad personality traits

We used the Spanish version of the Dark Tetrad at Work Scale (DTW), developed by Thibault and Kelloway [41] and adapted by Fernández-del-Río and colleagues [34]. It is rated on a 5-point Likert scale, ranging from 1 (*strongly disagree*) to 5 (*strongly agree*). It measures narcissism (6 items, α = .60), Machiavellianism (4 items, α = .60), psychopathy (6 items, α = .75), and sadism (6 items, α = .75). Total scores were computed as the sum of the scores of each dimension.

#### Task performance, contextual performance, and counterproductive work behaviors

The traditional three dimensions of job performance were measured with the Individual Work Performance Questionnaire (IWPQ) [42] in its Spanish adaptation [25]. It is rated on 5-point Likert scale, ranging from 0 (*seldom*) to 4 (*always*) for task performance (5 items, α = .84) and contextual performance (8 items, α = .74), and from 0 (*never*) to 4 (*often*) for counterproductive work behaviors (5 items, α = .75). Total scores are computed estimating the mean value of each dimension.

#### Adaptive performance

This dimension of job performance was measured with a 7-item version of the scale developed by Marques-Quinteiro and colleagues [43] adapted to Spanish by Ramos-Villagrasa et al. [39]. The items are rated on a 7-point Likert scale ranging from 1 (*totally ineffective*) to 7 (*totally effective*). In our study, the observed Cronbach’s alpha was .89.

### Analyses

Firstly, we computed the descriptive statistics of the variables (mean, standard deviation). Secondly, associations were calculated using Pearson correlations for numerical variables and point-biserial correlations for sex and responsibility. Finally, predictive models of job performance were computed with hierarchical regression analysis, using sociodemographic variables in Step 1, Big Five personality traits in Step 2, Dark Tetrad traits in Step 3, and biodata in Step 4. All analyses were performed with SPSS v.26.

## Results

Descriptive statistics, reliabilities, and correlations of the study variables are presented in Table 3. Internal consistency coefficients ranged from .60 to .89.

**Table 3:** Descriptive statistics and bivariate relations of the study variables.

Variables	*M*	*SD*	α	Associations																		
				1	2	3	4	5	6	7	8	9	10	11	12	13	14	15	16	17	18	19
				Pearson Correlations																		
1. Biodata (rational)	4.24	1.94	.63																			
2. Biodata (quasi-rational)	0.85	0.31	.63	.98**																		
3. Neuroticism	30.87	7.37	.81	-.23**	-.21**																	
4. Extraversion	43.65	6.05	.77	.14*	.16*	-.25**																
5. Openness	39.05	6.96	.79	.15*	.17**	-.05	.33**															
6. Agreeableness	42.73	6.50	.70	-.09	-.09	-.23**	.26**	.05														
7. Conscientiousness	46.08	5.83	.77	.24****	.25**	-.44**	.26**	.21**	.41**													
8. Narcissism	17.62	2.97	.60	.22**	.22**	-.02	.10	.12	-.06	.05												
9. Machiavellianism	10.14	3.20	.60	-.14*	-.13*	.13*	-.11	-.05	-.30**	-.17**	.20**											
10. Psychopathy	9.36	3.01	.75	-.07	-.09	.14*	-.15*	-.08	-.32**	-.20**	.21**	.47**										
11. Sadism	7.24	2.27	.75	-.06	-.05	.04	-.17**	-.06	-.26**	-.19**	.19**	.27**	.51**									
12. Task performance	3.24	0.62	.84	.15*	.16**	-.21**	.10	.02	.13*	.40**	.01	-.04	-.18**	-.23**								
13. Contextual performance	2.69	0.79	.87	.32**	.35**	-.18**	.23**	.24**	.01	.28**	.23**	-.14*	-.17**	-.18**	.40**							
14. Counterproductive behaviors	1.03	0.66	.75	-.01	-.01	.37**	-.18**	-.11	-.38**	-.26**	.05	.28**	.25**	.25**	-.17**	-.14*						
15. Adaptive performance	37.86	6.32	.89	.36**	.39**	-.09	.11	.30**	.07	.27**	.24**	-.08	-.20**	-.21**	.31**	.41**	-.06					
16. Age (years)	39.79	14.04		.15*	.11	-.07	-.10	-.21**	.09	.12	.02	-.11	.02	.01	.11	-.05	-.25**	-.05				
17. Job experience (months)	211.44	158.43		.17*	.13*	-.04	-.07	-.15*	.07	.14*	.05	-.07	.06	.03	.09	-.05	-.17**	-.03	.89**			
				Point-Biserial correlations																		
18. Sex	.50	.50		-.17*	-.17**	.20**	-.01	-.08	.18**	.01	-.11	-.12**	-.17**	-.19**	.13*	-.12	-.10	-.11	-.02	-.14*		
19. Responsibility	.26	.44		.52**	.50**	-.07	.01	.03	.01	.17**	.21**	-.07	-.05	-.04	.12	.16**	.02	.23**	.17**	.21**	-.25**	

*Note*. *N* = 264 (Subsample 2). Sex: 0 = Men, 1 = Women; Responsibility: 0 = No, 1 = Yes.

* = *p* < .05.

** = *p* < .01.

Regarding job performance, both biodata scales were related to all its dimensions except for Counterproductive work behavior (*M*_r_ = .28, range [.15, .36] for rational scoring and *M*_r_ = .30, range [.15, .39] for quasi-rational scoring). Concerning personality, each trait of the Big Five and Dark Tetrad was related to at least two performance dimensions (*M*_r_ = .24, range [.13, .40]).

Focusing on the relationship between biodata and personality, all the Big Five traits except for agreeableness were associated with the two biodata scores (*M*_r_ = .19, range [.14, .24] for rational scoring and *M*_r_ = .20, range [.16, .25] for quasi-rational scoring). Regarding the Dark Tetrad, only narcissism and Machiavellianism were significantly related (*M*_r_ = .18, range [.14, .22] for rational scoring and *M*_r_ = .18, range [.13, .22] for quasi-rational scoring). All these correlations were small according to Cohen [44], suggesting that these specific biodata items do not overlap with personality.

Predictive models of job performance are displayed in Table 4 (using rational scoring) and Table 5 (using quasi-rational scoring). Task performance and counterproductive behaviors models presented the same outcome for both approaches: only the Big Five personality played a role in the predictive models. Conscientiousness explained 21.2% of the variance of task performance (β = .39, *p* < .001). The model of counterproductive behaviors explained 32.9% of the variance of task performance and had four predictors: age (β = -.44, *p* = .004), neuroticism (β = .32, *p* < .001), openness (β = -.20, *p* = .003), and agreeableness (β = -.31, *p* < .001).

**Table 4:** Regression analysis of job performance using rational biodata.

	Task performance			Contextual performance			Counterproductive behaviors			Adaptive performance		
	*R* ^2^	Δ*R*^2^	*p*	*R* ^2^	Δ*R*^2^	*p*	*R* ^2^	Δ*R*^2^	*p*	*R* ^2^	Δ*R*^2^	*p*
*Step 1*	.074		**.005**	.046		.059	.069		**.008**	.088		**.001**
*Step 2*	.212	.138	**< .001**	.166	.120	**< .001**	.329	.260	**< .001**	.204	.116	**< .001**
*Step 3*	.241	.029	.140	.258	.092	**< .001**	.359	.030	.071	.275	.071	**.002**
*Step 4*	.241	.000	.933	.272	.014	< .062	.366	.006	.180	.324	.049	**< .001**
*Coefficients Step 1*	*β*	*p*		*β*	*p*		*β*	*p*		*β*	*p*	
Sex^a^	**.17**	**.025**		-.10	.182		-.03	.679		-.11	.145	
Age	.27	.109		.13	.446		**-.47**	**.006**		-.04	.817	
Job experience	-.12	.470		-.23	.190		.26	.125		-.07	.679	
Responsibility^b^	.13	.066		**.16**	**.035**		.05	.505		**.25**	**.00**	
*Coefficients Step 2*	*β*	*p*		*β*	*p*		*β*	*p*		*β*	*p*	
Sex^a^	.12	.095		-.11	.164		-.07	.329		-.13	.078	
Age	.26	.117		.21	.211		**-.44**	**.004**		.06	.702	
Job experience	-.15	.349		-.28	.094		.23	.130		-.14	.391	
Responsibility^b^	.08	.256		.10	.145		.07	.279		**.20**	**.003**	
Neuroticism	.01	.891		.01	.994		**.32**	**< .001**		.08	.311	
Extraversion	.05	.538		.15	.058		-.02	.745		-.04	.620	
Openness	-.07	.302		.14	.057		**-.20**	**.003**		**.27**	**< .001**	
Agreeableness	-.03	.735		-.07	.368		**-.31**	**< .001**		-.01	.909	
Conscientiousness	**.39**	**< .001**		**.23**	**.007**		.07	.335		**.20**	**.013**	
*Coefficients Step 3*	*β*	*p*		*β*	*p*		*β*	*p*		*β*	*p*	
Sex^a^	.13	.079		-.13	.074		-.04	.575		**-.15**	**.044**	
Age	.22	.174		.13	.404		**-.39**	**.010**		.01	.988	
Job experience	-.11	.505		-.20	.226		.18	.227		-.07	.656	
Responsibility^b^	.07	.360		.03	.642		.09	.185		**.14**	**.043**	
Neuroticism	.01	.917		-.02	.832		**.32**	**< .001**		.07	.334	
Extraversion	.02	.789		.10	.166		-.01	.845		-.07	.316	
Openness	-.08	.253		.10	.145		**-.19**	**.004**		**.23**	**.001**	
Agreeableness	-.04	.645		-.14	.066		**-.26**	**< .001**		-.06	.420	
Conscientiousness	**.38**	**< .001**		**.20**	**.013**		.09	.244		**.19**	**.016**	
Narcissism	.13	.058		**.24**	**.001**		.03	.702		**.25**	**< .001**	
Machiavellianism	.08	.322		-.14	.080		.11	.136		-.09	.223	
Psychopathy	-.07	.401		-.06	.483		-.10	.910		-.13	.124	
Sadism	-.11	.143		**-.20**	**.010**		.12	.076		-.07	.374	
*Coefficients Step 4*	*β*	*p*		*β*	*p*		*β*	*p*		*β*	*p*	
Sex^a^	.13	.080		-.13	.069		-.04	.564		**-.15**	**.034**	
Age	.22	.175		.14	.399		**-.39**	**.010**		.01	.955	
Job experience	-.11	.504		-.21	.202		.18	.243		-.09	.568	
Responsibility^b^	.06	.441		-.03	.671		.04	.559		.02	.813	
Neuroticism	-.01	.933		.01	.874		**.34**	**< .001**		.13	.091	
Extraversion	.02	.793		.10	.192		-.02	.795		-.09	.228	
Openness	-.08	.253		.09	.212		**-.20**	**.003**		**.20**	**.003**	
Agreeableness	-.03	.666		-.11	.161		**-.24**	**.001**		-.01	.979	
Conscientiousness	**.38**	**< .001**		**.19**	**.017**		.08	.276		**.18**	**.022**	
Narcissism	.13	.063		**.22**	**.002**		.01	.863		**.21**	**.002**	
Machiavellianism	.08	.321		-.12	.120		.12	.103		-.07	.389	
Psychopathy	-.07	.402		-.06	.490		-.01	.919		-.13	.120	
Sadism	-.11	.147		**-.19**	**.014**		.13	.061		-.05	.516	
Biodata	-.01	.933		.15	.062		.10	.180		**.28**	**< .001**	

*Note*. *N* = 264 (subsample 2).

^a^Coding: Men = 0, Women = 1;

^b^Coding: No = 0, Yes = 1.

Bold values correspond to statistically significant associations. Shaded cells belong to final predictive models.

**Table 5:** Regression analysis of job performance using quasi-rational biodata.

	Task performance			Contextual performance			Counterproductive behaviors			Adaptive performance		
	*R* ^2^	Δ*R*^2^	*p*	*R* ^2^	Δ*R*^2^	*p*	*R* ^2^	Δ*R*^2^	*p*	*R* ^2^	Δ*R*^2^	*p*
*Step 1*	.074		**.005**	.046		.059	.069		**.008**	.088		**.001**
*Step 2*	.212	.138	**< .001**	.166	.120	**< .001**	.329	.260	**< .001**	.204	.116	**< .001**
*Step 3*	.241	.029	.140	.258	.092	**< .001**	.359	.030	.071	.275	.071	**.002**
*Step 4*	.242	.001	.662	.279	.021	**< .020**	.366	.006	.180	.332	.057	**< .001**
*Coefficients Step 1*	*β*	*p*		*β*	*p*		*β*	*p*		*β*	*p*	
Sex^a^	**.17**	**.025**		-.10	.182		-.03	.679		-.11	.145	
Age	.27	.109		.13	.446		**-.47**	**.006**		-.04	.817	
Job experience	-.12	.470		-.23	.190		.26	.125		-.07	.679	
Responsibility^b^	.13	.066		**.16**	**.035**		.05	.505		**.25**	**.001**	
*Coefficients Step 2*	*β*	*p*		*β*	*p*		*β*	*p*		*β*	*p*	
Sex^a^	.12	.095		-.11	.164		-.07	.329		-.13	.078	
Age	.26	.117		.21	.211		**-.44**	**.004**		.06	.702	
Job experience	-.15	.349		-.28	.094		.23	.130		-.14	.391	
Responsibility^b^	.08	.256		.10	.145		.07	.279		**.20**	**.003**	
Neuroticism	.01	.891		.01	.994		**.32**	**< .001**		.08	.311	
Extraversion	.05	.538		.15	.058		-.02	.745		-.04	.620	
Openness	-.07	.302		.14	.057		**-.20**	**.003**		**.27**	**< .001**	
Agreeableness	-.03	.735		-.07	.368		**-.31**	**< .001**		-.01	.909	
Conscientiousness	**.39**	**< .001**		**.23**	**.007**		.07	.335		**.20**	**.013**	
*Coefficients Step 3*	*β*	*p*		*β*	*p*		*β*	*p*		*β*	*p*	
Sex^a^	.13	.079		-.13	.074		-.04	.575		**-.15**	**.044**	
Age	.22	.174		.13	.404		**-.39**	**.010**		.01	.988	
Job experience	-.11	.505		-.20	.226		.18	.227		-.07	.656	
Responsibility^b^	.07	.360		.03	.642		.09	.185		**.14**	**.043**	
Neuroticism	.01	.917		-.02	.832		**.32**	**< .001**		.07	.334	
Extraversion	.02	.789		.10	.166		-.01	.845		-.07	.316	
Openness	-.08	.253		.10	.145		**-.19**	**.004**		**.23**	**.001**	
Agreeableness	-.04	.645		-.14	.066		**-.26**	**< .001**		-.06	.420	
Conscientiousness	**.38**	**< .001**		**.20**	**.013**		.09	.244		**.19**	**.016**	
Narcissism	.13	.058		**.24**	**.001**		.03	.702		**.25**	**< .001**	
Machiavellianism	.08	.322		-.14	.080		.11	.136		-.09	.223	
Psychopathy	-.07	.401		-.06	.483		-.10	.910		-.13	.124	
Sadism	-.11	.143		**-.20**	**.010**		.12	.076		-.07	.374	
*Coefficients Step 4*	*β*	*p*		*β*	*p*		*β*	*p*		*β*	*p*	
Sex^a^	.13	.079		-.13	.072		-.04	.579		**-.15**	**.039**	
Age	.23	.168		.15	.348		**-.38**	**.012**		.03	.835	
Job experience	-.11	.493		-.21	.183		.17	.252		-.10	.518	
Responsibility^b^	.05	.537		-.05	.547		.04	.546		.01	.876	
Neuroticism	-.01	.980		.01	.860		**.34**	**< .001**		.12	.100	
Extraversion	.02	.806		.09	.203		-.02	.788		-.09	.209	
Openness	-.09	.236		.09	.231		**-.20**	**.002**		**.20**	**.004**	
Agreeableness	-.03	.723		-.10	.196		**-.24**	**.001**		.01	.946	
Conscientiousness	**.37**	**< .001**		**.18**	**.021**		.08	.294		**.17**	**.030**	
Narcissism	.13	.071		**.21**	**.002**		.01	.865		**.21**	**.002**	
Machiavellianism	.08	.310		-.12	.111		.12	.111		-.07	.335	
Psychopathy	-.07	.418		-.05	.574		-.01	.978		-.11	.179	
Sadism	-.11	.151		**-.19**	**.012**		.13	.066		-.05	.451	
Biodata	.04	.662		**.18**	**.020**		.10	.180		**.30**	**< .001**	

*Note*. *N* = 264 (Subsample 2).

^a^Coding: Men = 0, Women = 1;

^b^Coding: No = 0, Yes = 1.

Bold values correspond to statistically significant associations. Shaded cells belong to final predictive models.

With regard to contextual performance, the Big Five and the Dark Tetrad played a role in both predictive models, but biodata only did so if the quasi-rational approach was used. With this scoring method, the predictive model explained 27.9% of contextual performance, with conscientiousness (β = -.18, *p* = .021), narcissism (β = .21, *p* = .002), sadism (β = -.19, *p* = .012), and biodata (β = .18, *p* = .020) as predictors.

Adaptive performance was the last dimension analysed. Both biodata scoring methods were involved in the predictive models, but the model including quasi-rational scoring explained slightly more variance of adaptive performance compared to rational scoring (33.2% vs. 32.4%, respectively, with weights β = .30, *p* < .001 vs. β = .28, *p* < .001). The remaining predictors were sex (β_rational_ = -.15, *p* = .034; β_quasi_ = -.15, *p* = .039), openness (β_rational_ = .20, *p* = .003; β_quasi_ = .20, *p* = .004), conscientiousness (β_rational_ = .18, *p* = .022; β_quasi_ = .17, *p* = .030), and narcissism (β_rational_ = .21, *p* = .002; β_quasi_ = .21, *p* = .002).

## Discussion

Biographical information has many advantages that has made it a useful tool for personnel selection, especially thanks to the development of e-recruitment and application forms. However, issues regarding its content, scoring, and availability hinder further development both in research and professional fields. In this regard, García-Izquierdo and colleagues [14] took a step forward, elaborating and proposing a biodata scale for the evaluation of managers in public administration. The present research goes one step further, analyzing the biodata according to different job performance criteria, and with two different scoring methods (rational and quasi-rational biodata). Thus, the contribution of this study to scientific progress consists of: (1) providing evidence of the predictive validity of the biodata scale in a multi-occupational sample, also showing that the biodata scale can be used even with workers that do not hold a managerial position; (2) identifying that this biodata contributes to predicting two specific types of job performance: contextual performance and adaptive performance; (3) showing that a brief job-related biodata scale achieves results comparable to those of most personality traits in predictive models of two job performance dimensions (contextual and adaptive performance); and (4) providing evidence of the incremental predictive validity of biodata over the Big Five and the Dark Tetrad in the same performance dimensions. All of these results are discussed below.

First, the present study contributes to fulfilling one of the limitations pointed out by the developers of the scale: our findings show that their biodata scale is related to job performance. In addition, our data also show that these items can be useful in various types of jobs in different organizations, not only in public company management positions. This is reasonable, given that the content of biodata items, although related to managing staff, also refers to the typical behavior of a person with high conscientiousness, which is also associated with performance. In fact, the highest association of the biodata occurs with this personality trait. These biographical facts can also be related to self-leadership, which, in turn, is linked to performance.

Second, our results also show that the biodata analyzed are only involved in the prediction of contextual performance and adaptive performance. Concerning contextual performance, this may be due to the incorporation of non-managers in the study. The content of the biodata analyzed refers to improvements made, using person-related competences such as communication and negotiation, and being able to solve problems, which are behaviors related to supporting the organizational environment in non-managerial jobs. Thus, in these types of jobs, this description is better suited for contextual performance than for task performance [45]. This explains why in our study, we found a relationship with contextual but not with task performance. In any event, further research at the post-level may help to verify our explanation. Regarding adaptive performance, this is an interesting result, as, to our knowledge, this is the first study to relate biodata to this type of behavior at work. Further research should verify whether this relationship is specific to these items or is a common feature of biodata scales.

Third, this study shows that using a small set of job-related, mostly verifiable, and specific items we may have a useful biodata measure. It is not necessary to adopt a broader and probably less construct-validity perspective to have an adequate instrument. Our findings contribute to delimiting the biodata construct as separate from personality.

Lastly, the present study also contributes to the debate on the biodata construct itself by showing the differential role of biodata in predictive models, even considering two different sets of personality traits: the Big Five and the Dark Tetrad. Investigating this issue is relevant to keep the selection process simple, avoiding redundancy. Moreover, the biodata scale has shown similar results to those of the personality traits, somewhat in line with previous research [18].

### Limitations and further research

We acknowledge some limitations in this study. The main one is the use of self-report measures to assess job performance. However, we think that the advantages offered by this type of data, including confidentiality [46], have facilitated the collection of information and the ability to assess various types of performance simultaneously, including counterproductive behaviors that are often difficult to collect [47]. Further research may provide other types of criterion measures, such as supervisor ratings or objective data. Another noteworthy limitation is observed reliability: although all scales are above minimum standards, biodata, narcissism and Machiavellianism has values below .70. In the case of biodata scale, this may be due to its items measures different domains [13]. Regarding dark traits, the original version of the DTW demonstrate adequate values of test-retest reliability [41], but other studies carried out with the Spanish version narcissism report values around .60 and Machiavellianism around .75 [34, 35, 39]. Thus, we believe that the Machiavellianism’ observed reliability may be due to sample characteristics’ but for narcissism scale is related with the Spanish version of the DTW. Further research interested in narcissism may explore the use of more reliable measures.

Last but not least, this research did not consider whether the participant worked in a public or private organization. The biodata analysed in this paper were initially designed for the public domain, and we did not considered the nature of the organization in our analyses. Future research could examine whether this biodata works better in public than in private organizations.

Further research could analyze the relationship between this biodata scale and cognitive ability. Some authors have found a relationship between biodata and cognitive ability [e.g., 5, 10], but the items developed following the recommendations for previous research [12, 22] should avoid redundancy with cognitive ability. We expect that the present biodata reports similar results with cognitive ability than with personality.

### Implications for practitioners

Considering the above-mentioned limitations, we remark the following implications of the present study for professional practice: we recommend the use of this 7-item biodata scale, composed of job-related items that are mostly verifiable. It is easy to use and correct and, given their response format, they can be easily incorporated into the application forms of job-search websites (e.g., Infojobs, Monster), social networks (e.g., LinkedIn), or company websites. In addition, they can be used to evaluate candidates for a wide range of jobs, but only if we are interested in predicting contextual and adaptive performance. Besides that, previous research has found that the scale enacts positive reactions in practitioners and applicants [14]. Finally, we recommend their use even if we also plan to assess personality because they have shown incremental validity over the Big Five and Dark Tetrad personality traits.
